# Effects of propofol, ketamine-propofol mixture in pediatric dental patients undergoing intravenous sedation: a clinical study

**DOI:** 10.1038/s41598-024-61823-8

**Published:** 2024-05-23

**Authors:** Gizem Isık, Nilgun Alpay, Gülcin Daglioglu, Volkan Ciftci

**Affiliations:** 1 Department of Pediatric Dentistry, Faculty of Dentistry, Cukurova University, Sarıçam, 01330 Adana, Turkey; 2 Department of Anesthesiology and Reanimation, Faculty of Dentistry, Cukurova University, Adana, Turkey; 3 Department of Biochemistry, Balcalı Hospital Central Laboratory, Cukurova University, Adana, Turkey

**Keywords:** Anxiety, Ketofol, Intravenous sedation, Propofol, Salivary cortisol level, Health care, Health occupations, Medical research

## Abstract

This study aimed to evaluate the clinical effects, complications (peri- and postoperative), depth of sedation, recovery times, and changes in anxiety levels in paediatric dental patients receiving intravenous sedation with propofol and ketamine–propofol mixtures. This prospective clinical study included 69 healthy children (ASA 1) aged 3–7 years. The patients were assigned randomly to propofol group (n = 23), which received propofol; 1:3 ketofol group (n = 23), which received 1:3 ketofol; or 1:4 ketofol group (n = 23), which received 1:4 ketofol. The bispectral index (BIS) and Ramsay Sedation Scale (RSS) score were recorded at intervals of 5 min to measure the depth of sedation, and vital signs were evaluated. Peri- and postoperative complications and recovery times were recorded. Anxiety levels were also evaluated using the Facial Image Scale (FIS) and changes in saliva cortisol levels (SCLs) before and after the intravenous sedation procedure. The Kruskal‒Wallis test and Wilcoxon signed-rank test were used to determine pre- and posttreatment parameters. Dunn’s test for post hoc analysis was used to determine the differences among groups. Children’s pre- and posttreatment anxiety levels did not differ significantly according to FIS scores, and increases in SCLs were detected in 1:3 ketofol and 1:4 ketofol groups after dental treatment was completed. Compared with those in the other groups, the BIS values of the patients in 1:4 ketofol indicated a slightly lower depth of sedation. The recovery time of the patients in 1:3 ketofol was longer than that of patients in propofol and 1:4 ketofol. The incidence of postoperative complications (agitation, hypersalivation, nausea/vomiting, and diplopia) did not differ among the groups. Ketamine–propofol combinations provided effective sedation similar to that of propofol infusion without any serious complications during dental treatment performed under intravenous sedation. The ketofol infusion increased the anxiety level of paediatric dental patients to a greater extent than the propofol infusion.

## Introduction

Dental anxiety and its implications cause serious problems that negatively affect the oral health of children due to the avoidance of dental care^1,2^. Children's fear of dental treatment may lead to behaviour management problems for dentists, which can be a barrier to the successful comprehensive dental treatment of children. Sedation procedures can be used to relieve anxiety and manage behaviour in anxious children undergoing dental treatment^3,4^. In children exhibiting high anxiety levels, these sedation procedures are used to perform dental treatment when a psychological approach and basic behaviour management techniques are insufficient.

Many sedative agents can be administered intravenously (IV) for procedural sedation and analgesia (PSA) in paediatric dental practice. Propofol, first approved by the Federal Drug and Administration (FDA) in 1989 as an anaesthetic, has been used in IV applications for multiple indications, including aesthesia and sedation. Propofol is a short-acting injectable anaesthetic medication composed of 2,6-diisopropylphenol^5^. The alkylphenol derivative propofol is used to sedate patients and induce and maintain anaesthesia throughout medical operations. The agonistic effects of propofol on the gamma-aminobutyric acid receptor are linked to its pharmacologic mechanism^6,7^. The strong affinity of propofol for gamma-aminobutyric acid receptors results in a notable analgesic effect^8^. Furthermore, propofol blocks the baroreceptor reflex and reduces sympathetic activity. Moreover, propofol increases the synthesis of nitric oxide, which results in vasodilation. However, the use of propofol can be associated with insufficient analgesia and an increased incidence of side effects when excessive propofol doses are administered or when propofol is coadministered with opioids^9^*.* A study showed that the use of propofol in the emergency department (ED) is a likely cause of cardiorespiratory depression^10^.

Ketamine is a derivative of phencyclidine that was first produced in 1962 and is widely used for PSA and to induce and maintain anaesthesia. N-Methyl-D-aspartate, opioid, and monoaminergic receptors are among the receptors affected by ketamine. High ketamine concentrations inhibit muscarinic receptors and promote neurotransmission via gamma-aminobutyric acid receptors^11^. Ketamine prevents patients from perceiving sensory cues by causing the cortex and limbic systems to dissociate. Ketamine is an analgesic and amnestic agent that maintains the throat reflex and increases blood pressure and myocardial oxygen demand by stimulating the cardiovascular system. Injectable, intramuscular, intradermal, oral, nasal, and rectal routes of ketamine administration are all used. Additionally, when diluted in a solution without preservatives, ketamine can be administered intrathecally or epidurally. The effectiveness of regional anaesthesia can be increased by using ketamine as an adjunct or supplement to local or regional anaesthetics. Furthermore, doses of 0.1–0.6 mg/kg are increasingly being used in the ED for brief, unpleasant procedures. While ketamine is a preferred agent with pharmacodynamic properties that preserves cardiac and respiratory functions in procedures that are painful and brief for dental sedation^12^, clinical guidelines have documented the risk of laryngospasm, which has increased in recent years^13^.

Propofol infusion can cause respiratory depression at high doses. Although the dissociative agent ketamine also maintains cardiopulmonary stability, its use can result in extended recovery, emesis, hypersalivation, and hallucinations. Propofol alone rapidly penetrates the blood–brain barrier to enter the central nervous system and provides rapid sedation with a short recovery period, but its use increases the risk of hypotension. The addition of ketamine minimizes the risks of respiratory depression and haemodynamic changes. Ketamine also has analgesic properties to supplement the anaesthetic properties of propofol. The combination of the 2 medications allows the use of lower doses of each agent, which leads to respiratory and haemodynamic stability while preserving the anaesthetic or analgesic properties that each drug offers.

Theoretically, combining propofol and ketamine could preserve sedation efficacy while minimizing the risk of adverse cardiovascular effects through dose reduction. The aim of the use of a combination of ketamine and propofol (ketofol) is to benefit from the synergistic interaction of these two drugs. Ketofol is used to provide PSA for procedures in the ED and hospital. In a case series, the use of a subdissociative dose of ketamine did not lead to any additional respiratory protection when combined with propofol^14^. A clinical study showed that the incidence of side effects decreased as the dose decreased during treatment with the combination of the two drugs^15^.

The use of ketofol by mixing ketamine and propofol in a single syringe is new in dental practice. Ketofol is purported to reduce respiratory depression, emesis, and the recovery time by counteracting the negative effects of one drug with the positive effects of the other. Some studies have documented that the successful use of ketofol for nondental procedures varies considerably in terms of dose and side effects^16,17^, but less is known about the potential benefits and risks of using a combination of sedative agents used in dental sedation procedures^18^ and its effects on the cortisol system. This study aimed to evaluate the clinical effects, peri- and postoperative complications, sedation depth, and recovery time of children receiving intravenous sedation with ketamine–propofol mixtures (1:3 and 1:4) and to compare them to those of children receiving propofol infusion as a control group and to evaluate the changes in anxiety levels of the children before and after these regimens by measuring the salivary cortisol level (SCL) and Facial Image Scale (FIS) score. According to the null hypothesis, H_0_, IV sedation would cause a nonsignificant increase in anxiety levels in paediatric dental patients.

## Materials and methods

### Study design

The study was designed as an observational clinical trial and was performed at the Cukurova University Faculty of Dentistry, Paediatric Dentistry Department. All the experimental protocols were approved by the Institutional Ethical Committee of Cukurova University (02/11/2018 82/2), registered on ClinicalTrial.gov (NCT04623970-10/11/2020), and performed in accordance with the guidelines on human studies outlined in the Declaration of Helsinki.

### Selection of participants

This study included healthy paediatric patients aged 3–7 years in the American Society of Anaesthesia (ASA) I risk group with no motor deficits, mental retardation, previous sedation or general anaesthesia history who were admitted to the Cukurova University Faculty of Dentistry, Department of Paediatric Dentistry, for dental treatment. We evaluated the sample sizes of previous studies^17,20,21^ that we found to have the most comparable study design to ours, and we determined our sample size using G*Power software. Seventy-two participants were included in the study to perform a comparable analysis with an alpha level of 0.05 and a test power of 88%.

Patients who had no previous dental treatment and had a high level of anxiety according to Frankl’s Behaviour Rating Scale^22^ (FBRS), which is an ordinal scale with 4 ratings used to describe a child’s behaviour [definitely negative (1), negative (2), positive (3), and definitely positive (4)], were included in the study. In the present study, the FBRS was administered at the patients’ first dental visit, and patients with FBRS scores ≤ 2 were randomly distributed to the study groups. Children who had upper or lower respiratory tract infections, cold or flu symptoms, or who used any medication on the day of the operation or 48 h before for any reason were excluded from the study. Any child who used a drug in the last 6 months that affected the composition of saliva or who had a disease that affected the functioning of the salivary glands and made it difficult to obtain a saliva sample was also excluded.

Seventy-two patients (35 females and 37 males) were divided into 3 sedative agent groups using a random sequence generator (computer program) at the RANDOM.ORG website. However, during the postoperative analysis of the samples, 3 patients were excluded from the study because the preoperative saliva sample was insufficient for two patients in propofol and 1:4 ketofol groups, and the postoperative saliva sample was insufficient for one patient in 1:3 ketofol group. The study therefore included 69 patients (32 females and 37 males) who were assigned to propofol (n = 23), 1:3 ketofol (n = 23), or 1:4 ketofol (n = 23) groups.

### Interventions

The children were physically examined by an anaesthetist before the procedures. Written consent was obtained from the children’s guardians. The anaesthetist stressed that the child should not consume solid or liquid food for at least 8 h before the operation. One day before the planned treatment and on the day of the procedure itself, parents were instructed not to brush their child’s teeth to avoid affecting the saliva composition. On the operation day, the patients were taken to a preoperation room together with their parents where they could play with toys and no dental equipment was present. The Facial Image Scale^23^ (FIS), which comprises a row of five faces ranging from ‘very unhappy’ to ‘very happy’ and numbered from 5 to 1, was used to determine the preoperative anxiety levels of the children. Subsequently, a minimum of 0.5 mL of unstimulated saliva was collected to measure preoperative cortisol levels by placing the cotton pellet from a salivary cortisol set (Salivette®, Sarstedt, Nümbrecht, Germany) on the tongues of the children and telling them not to chew for 1 min in accordance with the manufacturer’s instructions. These preoperative saliva samples were collected, placed in tubes and stored in a deep freezer at − 20 °C until analysis.

Twenty minutes before venous cannulation, oral midazolam at a dose of 0.5 mg/kg (Zolamid® 15 mg/3 mL IV) was administered to all children, and a mixture of 5% sevoflurane and 50% N_2_O–50% O_2_ was administered in the operating room via nasal mask before the dental procedures while children were under IV sedation to ensure standardization. The patients were cannulated, and the sedative drugs were administered according to the group assignment from the randomization chart.

*Propofol group:* Propofol (200 mg; Propofol® 1%, Fresenius, 10 mg/mL) was placed in the infusion device (Aitecs 2016, MOOG, USA), and a 20–60 µg/kg/min infusion was administered via the IV route. *1:3 ketofol group:* 50 mg ketamine (Ketax®, 500 mg/mL, 10 ml, Vem/Turkey) and 150 mg of propofol were combined, and the mixture was placed in the infusion device. An infusion of 20–60 µg/kg/min was administered via the IV route. *1:4 ketofol group:* 50 mg ketamine and 200 mg of propofol were drawn into a 20 cc injector. An infusion of 20–60 µg/kg/min was administered via the IV route.

### Sedation procedure and outcome measures

Topical anaesthesia (Xylocaine®-Pumpspray) was applied to the oral mucosa of all the teeth to be treated before the local anaesthesia procedure. Articaine hydrochloride (Ultracain® D-S Forte, Aventis) was used for all treated teeth. At least one of the following dental procedures was performed: pulpectomy, tooth extraction, pulpotomy, or restorative treatment. A medical nurse who was blinded to the drug administration protocol joined the paediatric dentist in performing the dental treatment procedures and monitored and recorded medical aspects of the sedation to avoid bias. All the dental procedures were planned to be completed within 45 min. During the sedation and dental procedures, the patients’ oxygen saturation (SpO_2_), pulse rate (PR), and systolic and diastolic arterial pressure (SAP/DAP) data were recorded electronically at 5-min. intervals. A decrease in SpO_2_ below 94% in patients was defined as desaturation. A pulse rate (PR) ≤ 60 bpm was considered to indicate bradycardia, and a pulse rate ≥ 140 bpm was considered to indicate tachycardia. In addition, the depth of sedation was recorded at 5-min intervals using bispectral index (BIS) monitoring with BIS paediatric Quattro sensors during the procedures. According to the manufacturer’s instructions (BIS® XP Aspect, Medical Systems, Norwood, MA, USA), a BIS value > 90 indicated an awake status; 71–90 indicated mild to moderate sedation; 61–70 indicated deep sedation; and 40–60 indicated general anaesthesia. The duration of the treatment, complications, and all dental treatment modalities were recorded on the patients’ follow-up form. The recovery status was assessed using the Modified Vancouver Sedation Recovery Scale (MVSDS). Patients were discharged when the MVSDS score was 1. Postoperative complications were evaluated using the Modified Aldrete Scale^24^ in the Post-Anaesthesia Care Unit (PACU), and recovery times were also recorded in the follow-up forms.

### Behaviour and dental anxiety analyses

The pre- and postoperative anxiety levels of all patients were assessed using the FIS and SCLs. The levels of anxiety were scored from 1 to 5 using the FIS at baseline (before sedation) and at the end of the treatment. Furthermore, saliva samples were collected again after the procedure using cotton pellets, which were then transferred to tubes from the SCL saliva kit (Salivette®, Sarstedt Inc., Nümbrecht, Germany). Saliva samples were collected at two different time intervals: before drug administration and after dental treatment. Saliva samples were collected 25 min after the procedure to evaluate the changes in anxiety levels in patients during the postoperative period. All saliva samples from the patients were collected between 09:00 and 12:00 AM to minimize the effect of diurnal variations in salivary cortisol levels.

### Analysis of saliva cortisol levels

The collected cotton pellets with saliva samples were stored in a deep freezer before enzyme-linked immunosorbent assay (ELISA). After the samples were thawed on the day of analysis, they were centrifuged at 4000 rpm for 10 min. After centrifugation, the obtained samples were analysed using a saliva cortisol analysis kit (Cortisol Saliva ELISA, Diametra, Italy) to measure the SCLs.

### Statistical analysis

Categorical measurements are summarized as numbers and percentages, continuous measurements are summarized as medians, Q1–Q3 (25th–75th percentiles), and means (minimum–maximum where appropriate). The chi-square test or Fisher’s exact test was used to compare categorical variables where appropriate. The Shapiro‒Wilk test was used to determine whether the parameters in the study showed a normal distribution. In the comparison of continuous measurements among groups, the Kruskal‒Wallis test was used for the parameters that did not show a normal distribution. Dunn’s test was used for post hoc tests after the K–W test, and adjusted p values are reported. The Wilcoxon signed-rank test was used to determine the differences between the pre- and posttreatment FIS scores and SCLs. Additionally, the differences (Δ) were calculated, and the median values of the differences were compared among groups. The statistical significance level was set at 0.05 for all tests. The IBM SPSS 23 (Armonk, NY: IBM Corp.) package was used for the statistical analysis of the data.

### Human rights

All procedures performed in this study involving human participants were in accordance with the ethical standards of the institutional and/or national research committee and with the 1964 Helsinki Declaration and its later amendments or comparable ethical standards.

### Informed consent

Informed consent was obtained from the participant's parents included in the study.

### Clinical relevance

Knowing which sedative agent and/or combination of IV sedation agents used in dental procedures for anxious children will have more efficient clinical and positive effects on anxiety.

## Results

**Figure 1:**
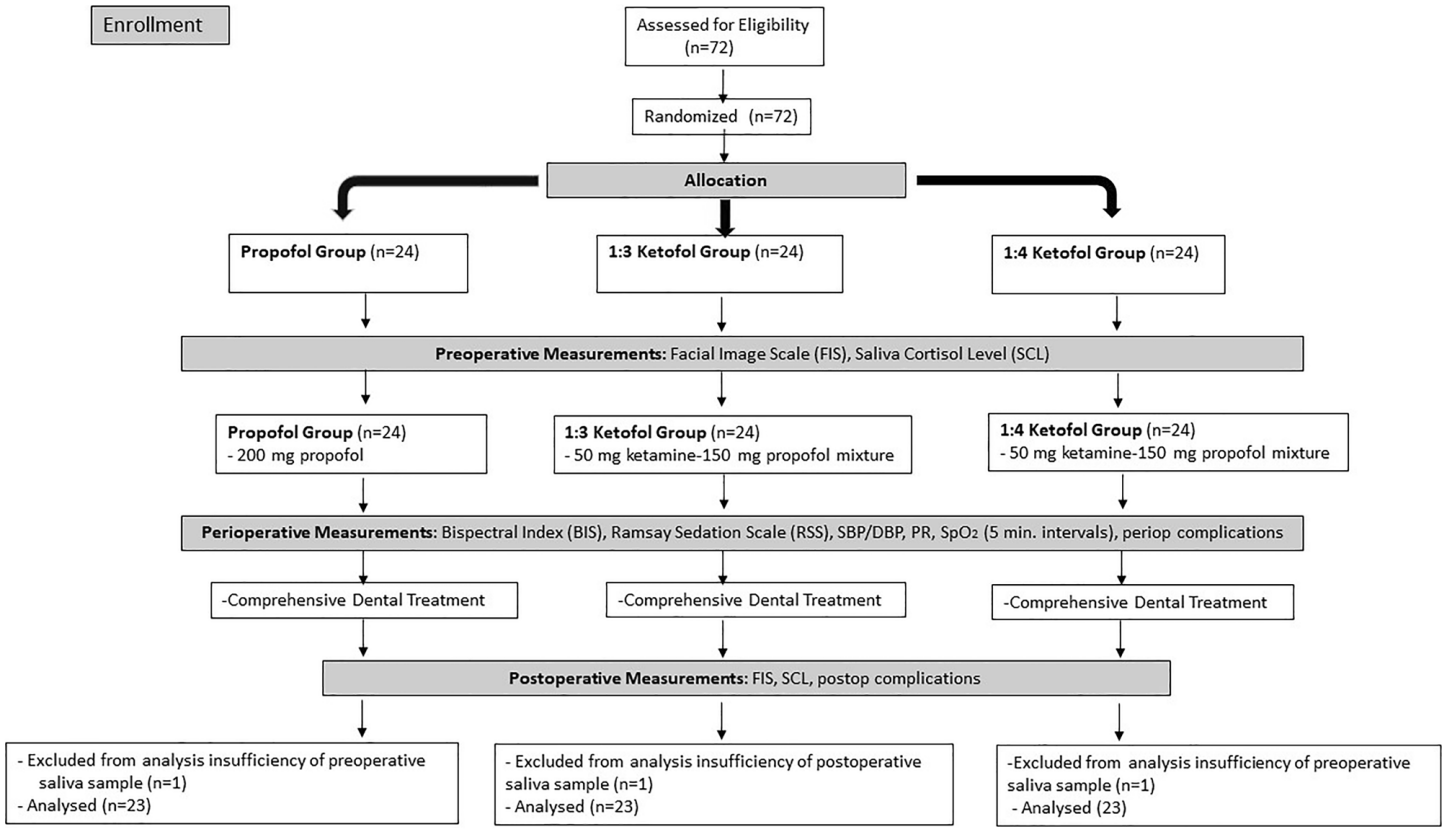
The flowchart showing the enrollment, allocation, and analysis of subjects involved in the study.

Sixty-nine paediatric patients who underwent IV sedation during comprehensive dental treatment were included in the study The flowchart shows the enrollment, allocation, and analysis of patients involved in the study (Fig. 1). When the groups were assessed according to demographic data such as age, sex, weight, and the Frankl Behaviour Rating Scale score, a statistically significant difference was not observed among the groups. The baseline characteristics of all patients are summarized in Table 1.

**Table 1:** The baseline demographic characteristics of the patients.

	Propofol	1:3 Ketofol	1:4 Ketofol	*p* value
Age (years)	5.0 (3.5–7.0)	4.5 (3.0–6.5)	4.9 (3.5–7.0)	0.281*
Female	12 (52.2%)	10 (43.5%)	10 (43.5%)	0.792^+^
Male	11 (47.8%)	13 (56.5%)	13 (56.5%)	
Weight (kgs)	18.7 (14.0–33.0)	17.9 (15.0–22.0)	18.0 (12.0–33.0)	0.769*
FBRS score	1.73 (1.0–2.0)	1.70 (1.0–2.0)	1.7 (1.0–2.0)	0.741*

The results are presented as n (%) for sex and as the means (min–max) for age, weight and FBRS scores.

*FBSR* Frankl’s Behavior Rating Scale.

*Kruskal‒Wallis test, ^+^chi-square test.

**Figure 2:**
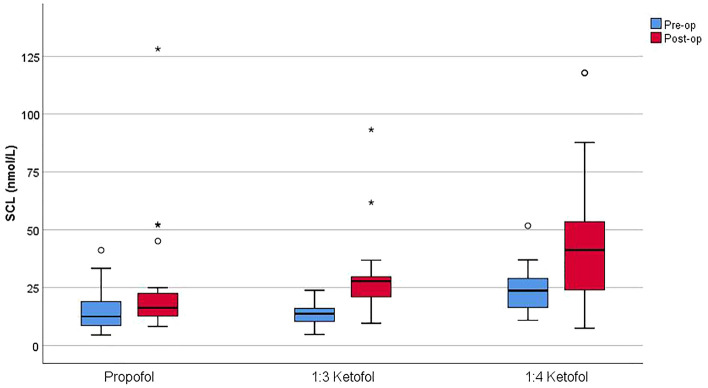
Comparison of saliva cortisol levels (SCL) among the groups.

When the patients’ anxiety levels were assessed using the FIS, the pre- and postoperative differences among the groups were not statistically significant (p = 0.091 and p = 0.558, respectively). Comparisons of anxiety levels using FIS scores are shown in Table 2. When preoperative and postoperative SCLs were compared, no significant difference was found in the propofol group (p = 0.094), whereas preoperative and postoperative SCLs were different in the 1:3 and 1:4 propofol groups (p < 0.001 and p = 0.001, respectively). When preoperative SCLs were compared among the three groups, a statistically significant difference was observed (p < 0.001). When the group from which the difference originated was investigated, the preoperative SCL was significantly greater in the 1:4 ketofol group than in the propofol and 1:3 ketofol groups (p = 0.002 and p = 0.002, respectively). Similarly, postoperative SCLs were significantly different among the groups (p = 0.001), and patients in the 1:4 ketofol group had higher postoperative SCLs than patients in the propofol group (p = 0.001). In the evaluation of the origin of the change, the differences between the postoperative and preoperative measurements (Δs) were calculated, and the median values of the differences were compared among the groups. The median difference in the propofol group was lower than that in the 1:3 and 1:4 propofol groups (p = 0.026 and p = 0.022, respectively). The results from the preoperative and postoperative SCL evaluations of all the groups are shown in Table 3 and Fig. 2.

**Table 2:** Comparison of behavioural anxiety levels using FIS.

	Propofol	1:3 Ketofol	1:4 Ketofol	*p* value*
Preop FIS score	1.78 (1–5)	2.30 (1–5)	2.56 (1–3)	0.091
Postop FIS score	2.39 (1–4)	2.52 (1–4)	2.08 (1–3)	0.558
*p* value (t)	0.080	0.626	0.272	

The results are presented as the means (min–max).

*FIS* facial ımaging scale.

*Kruskal‒Wallis test, (t) paired sample t test.

**Table 3:** Comparison of pre- and postoperative SCLs according to group.

	Propofol	1:3 Ketofol	1:4 Ketofol	*p* values*	Post hoc test
Preop SCL (nmol/L)	12.5 (8.2–19.9)	13.7 (9.9–16.8)	23.7 (16.1–29.8)	** < 0.001**	3–1; **p = 0.002** 3–2; **p = 0.002**
Postop SCL (nmol/L)	16.2 (12.4–22.7)	27.8 (20.4–29.9)	41.3 (22.2–59.7)	**0.001**	3–1; **p = 0.001**
*p* value**	0.094	** < 0.001**	**0.001**		
Δ (delta)	2.1 (1.6–9.6)	14.8 (7.7–18.7)	13.1 (2.5–33.4)	**0.033**	3–1; **p = 0.026** 2–1; **p = 0.022**

The results are presented as the medians (Q1–Q3).

*Δ (delta)* postop-preop difference, *SCL* saliva cortisol level.

*Kruskal‒Wallis test, Dunn’s test was performed as a post hoc analysis, **Wilcoxon signed-rank test.

Significant values are given in bold.

**Figure 3:**
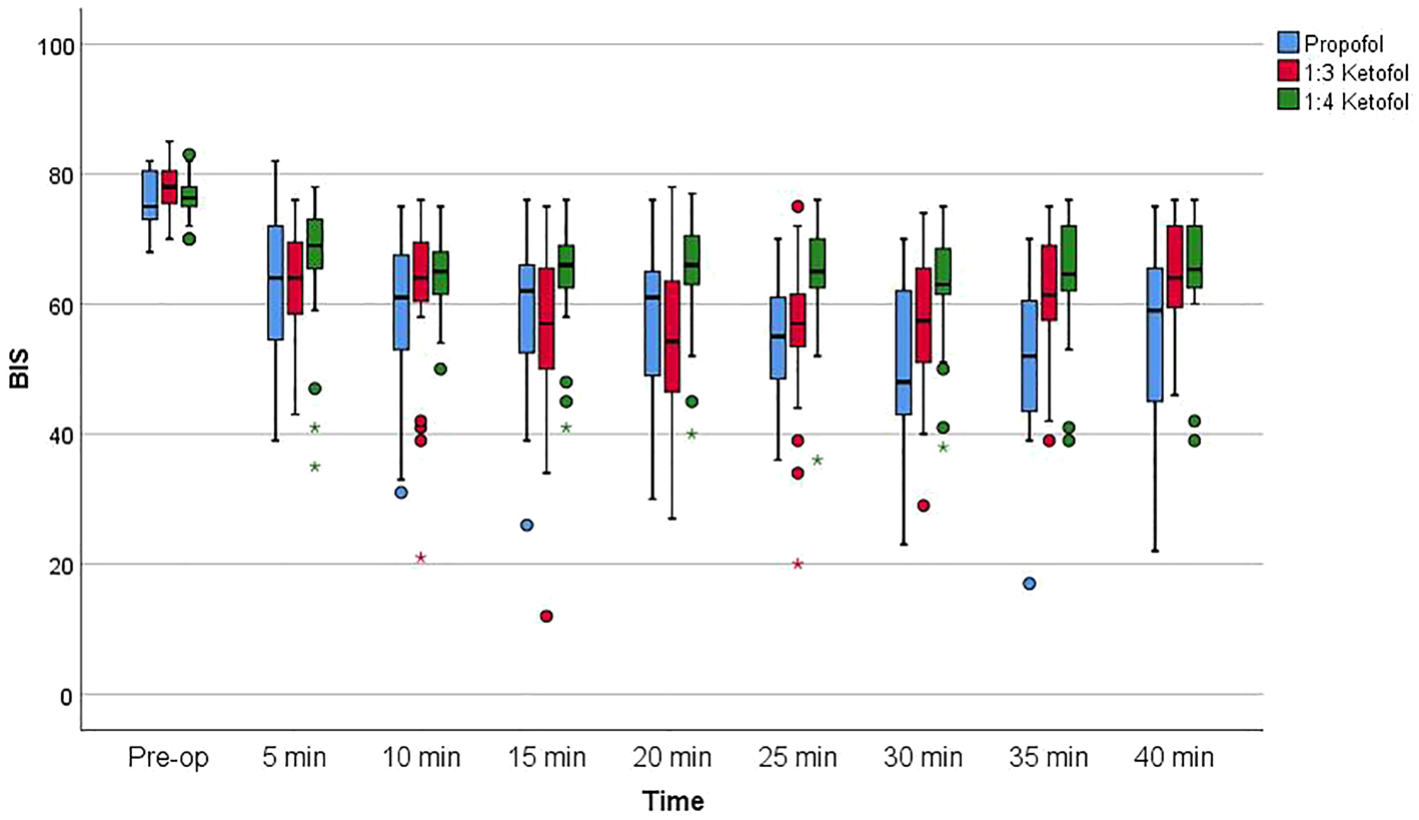
Comparison of bispectral ındex (BIS) among the groups.

When the BIS values at each time point were compared among groups, the values were similar at baseline and at 5, 10, and 15 min, whereas the differences in the scores at 20, 25, 30, 35, and 40 min were statistically significant. The results from the post hoc test of pairwise group comparisons at these time points are presented in Table 4 and Fig. 3. The RSS scores of the patients in the groups were also evaluated, and the scores at baseline were not significantly different from those observed 5, 10, 15, 20, 25, 30, 35 and 40 min later. The RSS scores of each group at the various intraoperative intervals are shown in Table 5.

**Table 4:** Comparison of BIS values at the indicated intraoperative intervals according to group.

	Propofol	1:3 Ketofol	1:4 Ketofol	*p* value*	Post hoc test
Preop	75.0 (73.0–81.0)	78.0 (75.0–81.0)	76.3 (75.0–78.0)	0.297	–
5 min	64.0 (53.0–72.0)	64.0 (56.0–70.0)	69.0 (65.0–74.0)	0.311	–
10 min	61.0 (53.0–69.0)	64.0 (60.0–71.0)	65.0 (60.0–68.0)	0.181	–
15 min	62.0 (51.0–67.0)	57.0 (48.0–66.0)	66.0 (62.0–69.0)	0.093	–
20 min	61.0 (48.0–65.0)	54.2 (45.0–64.0)	66.0 (63.0–72.0)	**0.005**	**3–2; p = 0.007**
25 min	55.0 (48.0–61.0)	57.0 (52.0–62.0)	65.0 (62.0–71.0)	** < 0.001**	**3–1; p = 0.031** **3–2; p = 0.002**
30 min	48.0 (43.0–63.0)	57.4 (49.0–67.0)	63.0 (61.0–71.0)	**0.007**	**3–1; p < 0.001**
35 min	52.0 (43.0–61.0)	61.4 (57.0–69.0)	64.6 (62.0–72.0)	** < 0.001**	**3–1; p = 0.005**
40 min	59.0 (45.0–66.0)	64.0 (59.0–73.0)	65.3 (65.3–62.0)	**0.009**	**2–1; p = 0.033** **3–1; p < 0.001**

The results are presented as the medians (Q1–Q3).

*BIS* bispectral index.

*Kruskal‒Wallis test, Dunn’s test was performed for a post hoc analysis.

Significant values are given in bold.

**Table 5:** Comparison of RSS values at the indicated intraoperative intervals according to group.

	Propofol	1:3Ketofol	1:4Ketofol	*p* value*
Preop	2.0 (2.0–2.0)	2.0 (2.0–2.0)	2.0 (2.0–2.0)	1.000
5 min	5.0 (4.0–5.0)	4.0 (4.0–5.0)	5.0 (5.0–5.0)	0.150
10 min	5.0 (5.0–5.0)	5.0 (4.0–5.0)	5.0 (5.0–5.0)	0.589
15 min	5.0 (5.0–5.0)	5.0 (5.0–5.0)	5.0 (5.0–5.0)	0.907
20 min	5.0 (5.0–5.0)	5.0 (5.0–5.0)	5.0 (5.0–5.0)	0.653
25 min	5.0 (4.0–5.0)	5.0 (5.0–5.0)	5.0 (5.0–5.0)	0.105
30 min	5.0 (5.0–5.0)	5.0 (5.0–5.0)	5.0 (5.0–5.0)	0.891
35 min	5.0 (5.0–5.0)	5.0 (5.0–5.0)	5.0 (5.0–5.0)	0.891
40 min	5.0 (5.0–5.0)	5.0 (5.0–5.0)	5.0 (5.0–5.0)	0.885

The results are presented as the medians (Q1-Q3).

*Kruskal‒Wallis test.

*RSS* Ramsey Sedation Scale.

**Figure 4:**
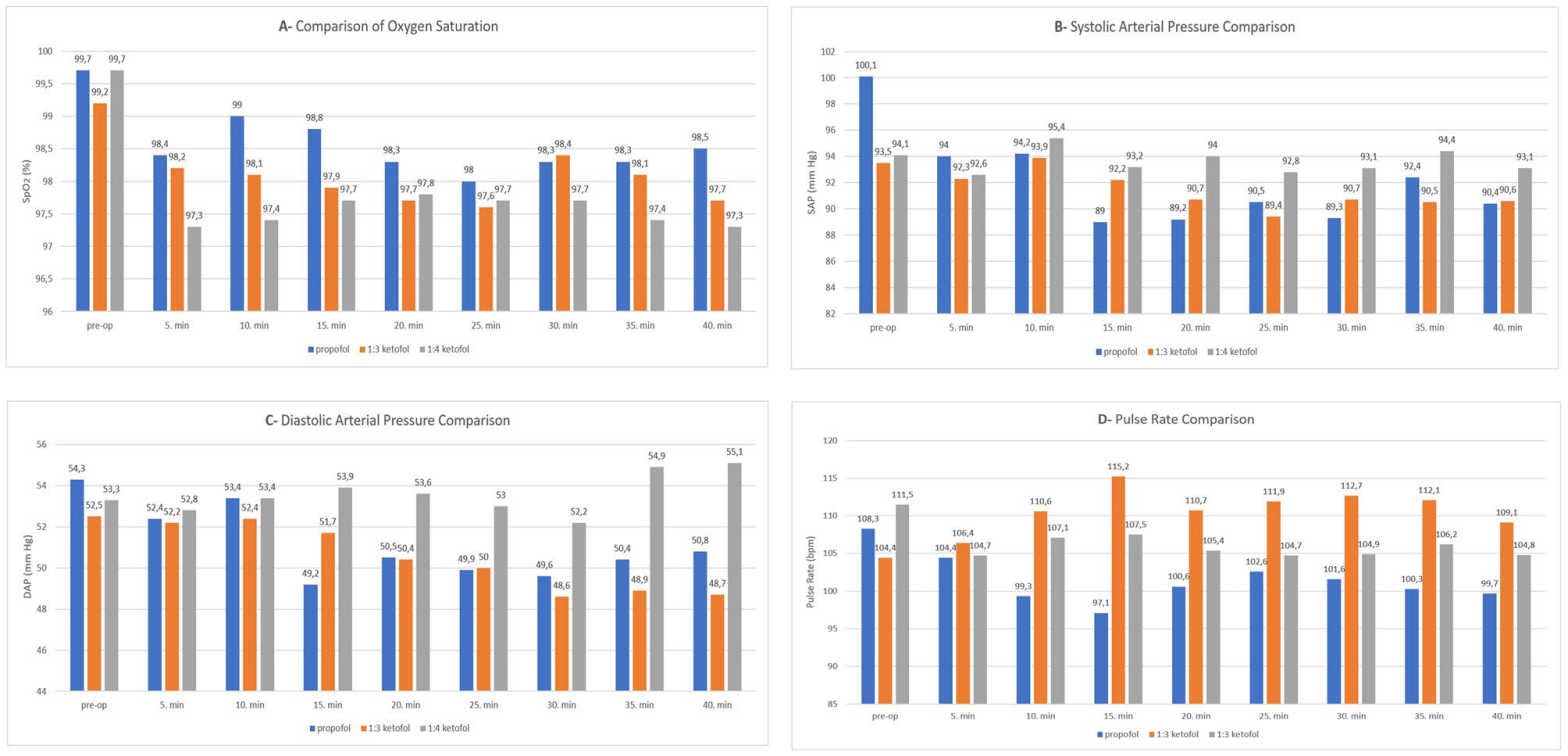
(**A**) Comparisons of oxygen saturation (SpO_2_), (**B**) systolic arterial pressure (SAP), (**C**) diastolic arterial pressure (DAP), and (**D**) pulse rate (PR) among the groups.

When the time-dependent change for the patients’ vital signs as oxygen saturation (SpO_2_), pulse rate (PR), and systolic and diastolic arterial pressure (SAP/DAP) were examined, the baseline values of all hemodynamic parameters were similar, and not found different from those observed 5, 10, 15, 20, 25, 30, 35 and 40 min later. The changes in the pulse rate (PR ≥ 60 bpm) did not last longer than 5 min in any patient. The oxygen saturation of patients was not decreased to below normal levels (SpO_2_ > 94%), and bradycardia or tachycardia were not observed. Comparisons of oxygen saturation, pulse rate, and systolic and diastolic arterial pressures were shown in Fig. 4A–D.

When complications in the perioperative period were examined, the presence of local anaesthesia (LA) injection pain did not differ significantly among the groups (p = 0.241). The incidence of coughing (p = 0.713), desaturation (p = 0.418) and spontaneous movement was not significantly different (p = 0.949). Postoperative complications (agitation, hypersalivation, cause/vomiting, and diplopia) were evaluated, and the incidence of complications did not differ among the groups (Table 6). The differences in recovery time among groups were significant (p < 0.001). The recovery time of patients in the 1:3 ketofol group (50 min) was significantly longer than the recovery times of patients in the propofol group (35 min) (p < 0.001) and the 1:4 ketofol group (35 min) (p = 0.002). The operation times were not different between the groups (average of 41 min). Comparisons of the perioperative and postoperative complications, operation times and recovery times among the groups are summarized in Table 6.

**Table 6:** Comparison of differences in the perioperative and postoperative complications, operating times, and recovery times between the groups.

	Propofol	1:3 Ketofol	1:4 Ketofol	p value***
Perioperative complications				
LA injection pain	15 (65.2%)	9 (39.1%)	10 (43.5%)	0.241
Coughing	5 (21.7%)	8 (34.8%)	7 (30.4%)	0.713
Desaturation	3 (13.0%)	4 (17.4%)	7 (30.4%)	0.418
Spontaneous movement	16 (69.6%)	15 (65.2%)	14(60.9%)	0.949
Postoperative complications				
Agitation	10 (43.5%)	14 (60.9%)	11 (47.8%)	0.567
Hypersalivation	0 (0.0%)	0 (0.0%)	0 (0.0%)	-
Nausea/vomiting	0 (0.0%)	1 (4.3%)	0 (0.0%)	0.999
Diplopia	0 (0.0%)	2 (8.7%)	1 (4.3%)	0.768
Recovery minutes	35.0 (30–40)	50.0 (35–60)	35.0 (25–45)	< 0.001*
Operating times	41.73 (40–45)	41.30 (40–45)	41.30 (40–45)	0.758

The results are presented as n (%) for categorical variables and as medians (Q1-Q3).

*LA* local anaesthesia.

*Kruskal‒Wallis test, ***Fisher’s exact test.

The number of restorative treatments and tooth extractions performed and their distribution among the groups were evaluated, and more restorative treatments were performed in the 1:4 ketofol group than in the propofol group (p = 0.007). A significantly greater number of extractions was performed in the propofol group than in the 1:3 ketofol group (p = 0.039). The results of the comparisons of the dental treatment modalities performed among the groups are summarized in Table 7.

**Table 7:** Comparison of the differences in dental treatment modalities between the groups.

	Propofol	1:3 Ketofol	1:4 Ketofol	P value*	Post hoc test
Restorative treatment	4.65 (1–10)	5.39 (2–11)	6.60 (3–11)	**0.007**	**3–1; p = 0.010**
Extraction	1.86 (0–6)	0.69 (0–4)	0.86 (0–3)	**0.039**	**1–2; p = 0.017**
Pulpectomy	0.21 (0–2)	0.13 (0–1)	0.34 (0–2)	0.312	
Pulpotomy	0.0 (0–0)	0.04 (0–1)	0.13 (0–1)	0.160	
Total	6.73 (3–12)	6.26 (3–13)	7.95 (4–13)	0.061	

The results are presented as the means (min–max).

*Kruskal‒Wallis test; Dunn’s test was performed as a post hoc analysis.

Significant values are given in bold.

## Discussion

The use of sedation could provide a suitable treatment environment for anxious children in paediatric dentistry. Sedation via the IV route was reported to be a more comfortable sedation protocol than sedation via other techniques^19^. The main disadvantage of using propofol alone in IV sedation is thought to be its propensity to lead to cardiorespiratory depression. Several factors are involved in this side effect, including opioid coadministration, the total dose of propofol, and the rate of infusion. In contrast, ketamine has advantages such as amnesia and consistent analgesia, cardiorespiratory safety, a short onset of action, rapid recovery time, and excellent potency with a unique dissociative effect, but laryngospasm, adverse airway effects (e.g., obstruction and hypoxemia) and agitation phenomena have been documented in the literature, and the increased risk has been notable in recent years^13^. In theory, replacing propofol with ketamine may preserve sedation efficacy while minimizing adverse cardiovascular effects by reducing the required dose and because of their similar effects. However, choosing which sedation agent is most reliable and efficient in children and is preferred for IV sedation procedures is very important.

Canpolat et al.^20^ used the FBRS to assess anxiety in their study of the use of ketamine, propofol, and 1:1 ketamine–propofol in children aged 3–9 years, and they found that ketamine increased dental anxiety to a greater extent than propofol and ketamine. A similar study using another anxiety scale revealed a decrease in the level of dental anxiety in both the propofol and 1:1 ketofol groups in children aged 6–12 years^21^. In this study, when the pre- and postoperative FIS scores of the groups were compared, no statistically significant differences were observed among the groups in terms of anxiety levels. The results of this study are not comparable to those of similar studies in the literature, as the use of subjective anxiety scales alone could not determine children’s anxiety levels and that the scores from these scales should also be supported by objective data.

Stress-related homeostasis is primarily regulated by the hormone cortisol. Saliva levels of the free form of cortisol increase significantly during physiological stress. Saliva cortisol levels are unaffected by the saliva volume and are strongly correlated with serum levels of the free form of cortisol. For healthy children, SCLs are between 5.52 and 28.92 nmol/L in the morning, while SCLs vary between 1.10 and 11.32 nmol/L in the afternoon^41^. A study also revealed that SCLs above 28 nmol/L are indicative of a high stress response in children^42^. Hsu et al.^43^ evaluated the effects of deep sedation on patients sedated for emergency procedures and reported that sedation itself is a stressful procedure and may cause a significant increase in SCLs. To the best of our knowledge, no other clinical study in the dental literature has evaluated the anxiety levels of children undergoing IV deep sedation with propofol and ketamine–propofol combinations (1:3 and 1:4) by measuring SCLs before and after dental procedures. In our study, the mean SCL before the dental procedures in the three study groups was less than 28 nmol/L. However, the postoperative SCLs in 1:3 ketofol (27.8 nmol/L) and 1:4 ketofol (41.3 nmol/L) were significantly higher than those in propofol group (16.2 nmol/L), and the mean postoperative SCLs were similar to or less than 28 nmol/L in patients in propofol (16.2 nmol/L) and 1:3 ketofol groups (27.8 nmol/L), indicating the absence of a high stress response. A study showed that chronic conditions involving the cortisol system can be found in patients receiving ketamine^44^. Another study revealed that even at low doses, ketamine doubles cortisol production^45^. The presence of a cortisol response has been suggested to merely be a biochemical phenomenon and is not of clinical importance in terms of minor surgeries (performed under ketamine) and mild stress. In our study, the pre- and posttreatment SCLs differed between the 1:3 and 1:4 ketofol groups (Δ = 14.8 and 13.1, respectively). We concluded that the 1:4 ketofol mixture caused a greater stress response than propofol and 1:3 ketofol in children after the IV sedation procedure. The presence of ketamine affected children’s anxiety levels. Therefore, our null hypothesis that sedation would cause a nonsignificant increase in anxiety levels in paediatric dental patients was rejected.

One factor affecting anxiety levels is the type of dental treatment performed. Miller et al.^25^ reported that tooth extraction increased cortisol levels. However, in this study, a greater number of extractions was performed in the propofol group than in the 1:3 and 1:4 ketofol groups, but fewer changes in the SCLs were detected after the dental procedure in the propofol group. This finding indicates that anxiety levels are independent of the dental procedure performed and supports our conclusion that the effects of sedation agents used for children's anxiety may be of greater importance.

Desaturation due to sedation agents is a serious complication in dental treatment performed under sedation. Since the oral cavity is the working area of dentists, the risk of the aspiration of saliva, blood, or dental material/equipment makes this complication even more important. Kip et al.^26^ evaluated the effects of different ketofol concentrations during dental treatments under sedation. They found a decrease in oxygen saturation in 5 patients in the study group treated with 1:1 ketofol, 2 patients treated with 1:2 ketofol, and 1 patient treated with 1:4 ketofol, but no statistically significant differences were observed in respiration or desaturation among their study groups. During endodontic treatment, Mittal et al.^27^ reported that oxygen saturation decreased in 9 patients in the ketofol group (1:4) and 3 patients in the propofol group. Singh et al.^28^ reported that patients in the propofol group had more respiratory complications during medical operations than did those in the ketofol group. In this study, oxygen saturation decreased in 7 patients in the 1:4 ketofol group, 4 patients in the 1:3 ketofol group, and 3 patients in the propofol group, and the incidence of this complication was significantly greater in the 1:4 ketofol group than in the other groups. The results of this study are in agreement with those of Mittal et al.^27^ A possible reason why the results of this study differ from those of studies in the field of medicine is that dental procedures are exclusively intraoral, and the instruments that we use for dental treatment are air‒water cooled. In addition, the increase in salivary secretion in the 1:4 ketofol group might also decrease saturation by affecting the associated airways.

A study showed that propofol sedation could lead to children experiencing more pain while undergoing certain potentially painful procedures, such as local anaesthesia (LA) injections^21^. Although LA injection pain was observed in our groups, 15 patients (65.2%) in the propofol group experienced more pain during LA injection than did those in the other groups. This finding is attributed to the fact that propofol alone does not relieve pain. Therefore, we must emphasize that propofol should be combined with an effective analgesic agent during potentially painful dental procedures such as tooth extraction and minor surgery. Andolfatto et al.^29^ reported that the number of spontaneous movements in the propofol group during treatment was greater than that in the ketofol group. No statistically significant difference in the number of spontaneous movements was observed among the groups in this study. We observed spontaneous movements in all three groups, with the greatest number observed in the propofol group (69.6%). This increased movement may be due to the children experiencing more LA injection pain in response to propofol because of the greater number of injections administered or the absence of pain-relieving properties of ketamine when administered alone.

In their study, Mittal et al.^27^ reported that cough developed in more patients in the ketofol group than in the propofol group, where they used propofol and 1:4ketofol. In this study, the frequency of cough was higher in the 1:3 ketofol group (34.8%) and 1:4 ketofol group (30.4%) than in the propofol group (21.7%), although the difference was not significant. We concluded that cough develops in all patients, regardless of the sedative agent used, due to the oral nature of the surgery and the use of air‒water-cooled instruments.

The bispectral index (BIS) is a monitoring system used to evaluate the depth of sedation. A few clinical studies have shown that a mean BIS value of 70 is sufficient to achieve moderate or deep sedation to provide dental treatment^30,31^. Eshghi et al.^32^ reported that a mean BIS value of 50.05 was obtained in their ketamine group 45 min after the start of the procedure. Cillo et al.^33^ evaluated propofol and ketofol with BIS monitoring during oral surgery and reported that the mean BIS values were 63.2 for propofol, 69.6 for 1:10 ketofol, 71.8 for 1:5 ketofol and 72.1 for 1:3 ketofol. They reported that as the dose of propofol in the ketofol mixture increased, the BIS value decreased. Kip et al.^21^ reported that the BIS value of ketofol at a ratio of 1:1 was similar to or lower than that of ketamine. In this study, significant differences in the mean BIS values among the groups were found. BIS values were higher in the ketofol groups than in the propofol group, especially after 35 min. In addition, the 1:4 ketofol group had slightly higher BIS values than the other groups at all time intervals. Although the sedation levels during all procedures were clinically acceptable, 1:4 ketofol provided a slightly lower depth of sedation than did the other regimens.

Some studies have reported that complications during the recovery period, such as agitation and diplopia, are psychomimetic effects caused by ketamine^34,35^. Other studies have documented that these psychomimetic effects are observed when ketamine is administered alone and are reduced when ketamine is combined with propofol^29,36^. Andolfatto and Willman^36^ reported agitation in only 2 patients during the postoperative period following sedation with ketofol in their study. Similarly, Kip et al.^21^ reported that the propofol group exhibited no agitation, but agitation was observed in 2 patients in the ketofol group. In this study, agitation was observed in all groups (n = 10, n = 14, and n = 11 in propofol, 1:3 ketofol and 1:4 ketofol groups, respectively), unlike in similar studies in the literature. DaSilva et al.^37^ and Shah et al.^38^ reported that only 2 patients experienced diplopia in their studies in which they evaluated ketofol sedation in children aged 2–17 years. In our study, diplopia was observed only in our ketofol groups (n = 2 and n = 1 in 1:3 ketofol and 1:4 ketofol groups, respectively), but these differences were not statistically significant.

daSilva et al.^37^ and Shah et al.^38^ observed no hypersalivation in any of their patients during the recovery period following sedation with ketofol. Similarly, hypersalivation was not observed in any of our groups. Additionally, Shah et al.^38^ reported that during the recovery period, nausea/vomiting was observed in 12% of patients in the ketamine group and 2% of patients in the ketofol group. Kip et al.^21^ reported that nausea and vomiting occurred in 5 patients (20%) in the ketamine group, 2 patients (8%) in the 1:1 ketofol group, and no patients in the propofol group. In our study, nausea/vomiting was observed in only 1 patient in the 1:3 ketofol group. Our results are in agreement with the results of similar studies in the literature.

In studies using ketamine, propofol, and 1:1 ketofol sedative regimens, Canpolat et al.^20^ reported that the recovery time of patients in the propofol group was significantly shorter than that of patients in the ketofol group. Similarly, Mittal et al.^27^ reported similar results in their study using propofol and 1:4 ketofol. In our study, we found that the treatment times (average of 41 min) were similar among the groups, but the recovery times were significantly different. The recovery times were shorter for propofol (35 min) than for 1:3 and 1:4 ketofol (50 min and 35 min, respectively). Daabis et al.^39^ evaluated the side effects of using 1:1 and 1:4 ketofol. They found that in the 1:1 ketofol group, which had a high ketamine ratio, ketamine prolonged the recovery period due to its delaying effect. In this study, when we compared the ketofol groups, we found that the recovery time of 1:3 ketofol group (50 min) was longer than that of 1:4 ketofol group (35 min), which was consistent with other studies in the literature.

The American Academy of Paediatric Dentistry (AAPD) has emphasized the necessity of monitoring vital signs for safe sedation practice since the morbidity rate due to sedation is higher in children than in adults^40^. In our study, in which the vital signs of patients in all groups were examined during the procedure, changes in the pulse rate (PR ≥ 60 bpm) did not last longer than 5 min in any patient. The oxygen saturation of patients never decreased to below normal levels (SpO_2_ > 94%), and bradycardia or tachycardia were not observed as complications, as the heart rate remained within acceptable limits^46^.

The present study has several limitations. Although the results observed and discussed are significant, our sample size is small. In future clinical studies, changes in anxiety levels and SCLs should be evaluated at different time intervals after intravenous dental sedation in larger sample sizes of patients receiving different sedation regimens.

## Conclusions

Based on our findings, we concluded that the use of propofol and ketamine–propofol in combination achieves effective deep sedation without any serious complications. Among the included patients, the haemodynamic stabilities and physiological parameters of the sedation regimens used for comprehensive dental treatment involving IV sedation were similar at various time points. However, the 1:4 ketofol sedation regimen led to a greater stress response than did the propofol and 1:3 ketofol regimens in paediatric dental patients. Additionally, the 1:3 ketofol sedation regimen was associated with a slightly longer recovery time but no high-stress response.

## Data Availability

The data that support the findings of this study are available from [corresponding author] but restrictions apply to the availability of these data, which were used under license for the current study, and so are not publicly available. Data are however available from the authors upon reasonable request and with permission of [corresponding author].
